# Tyr1068-phosphorylated epidermal growth factor receptor (EGFR) predicts cancer stem cell targeting by erlotinib in preclinical models of wild-type EGFR lung cancer

**DOI:** 10.1038/cddis.2015.217

**Published:** 2015-08-06

**Authors:** G Sette, V Salvati, M Mottolese, P Visca, E Gallo, K Fecchi, E Pilozzi, E Duranti, E Policicchio, M Tartaglia, M Milella, R De Maria, A Eramo

**Affiliations:** 1 Regina Elena National Cancer Institute, Rome, Italy; 2 Department of Hematology, Oncology and Molecular Medicine, Istituto Superiore di Sanità, Rome, Italy; 3 Department of Surgical Sciences, La Sapienza University of Rome, Rome, Italy; 4 Department of Experimental Medicine, La Sapienza University of Rome, Rome, Italy

## Abstract

Tyrosine kinase inhibitors (TKIs) have shown strong activity against non-small-cell lung cancer (NSCLC) patients harboring activating epidermal growth factor receptor (EGFR) mutations. However, a fraction of *EGFR* wild-type (WT) patients may have an improvement in terms of response rate and progression-free survival when treated with erlotinib, suggesting that factors other than EGFR mutation may lead to TKI sensitivity. However, at present, no sufficiently robust clinical or biological parameters have been defined to identify WT-EGFR patients with greater chances of response. Therapeutics validation has necessarily to focus on lung cancer stem cells (LCSCs) as they are more difficult to eradicate and represent the tumor-maintaining cell population. Here, we investigated erlotinib response of lung CSCs with WT-EGFR and identified EGFR phosphorylation at tyrosine1068 (EGFR^tyr1068^) as a powerful biomarker associated with erlotinib sensitivity both *in vitro* and in preclinical CSC-generated xenografts. In contrast to the preferential cytotoxicity of chemotherapy against the more differentiated cells, in EGFR^tyr1068^ cells, erlotinib was even more active against the LCSCs compared with their differentiated counterpart, acquiring potential value as CSC-directed therapeutics in the context of WT-EGFR lung cancer. Although tumor growth was inhibited to a similar extent during erlotinib or chemotherapy administration to responsive tumors, erlotinib proved superior to chemotherapy in terms of higher tolerability and reduced tumor aggressiveness after treatment suspension, substantiating the possibility of preferential LCSC targeting, both in adenocarcinoma (ADC) and squamous cell carcinoma (SCC) tumors. We conclude that EGFR^tyr1068^ may represent a potential candidate biomarker predicting erlotinib response at CSC-level in EGFR-WT lung cancer patients. Finally, besides its invariable association with erlotinib sensitivity in EGFR-WT lung CSCs, EGFR^tyr1068^ was associated with EGFR-sensitizing mutations in cell lines and patient tumors, with relevant diagnostic, clinical and therapeutic implications.

Non-small-cell lung cancer (NSCLC) accounts for ∼80% of lung cancer subtypes and is the leading cause of cancer-related death worldwide.^1^ In recent years, molecular characterization of NSCLC has reached an unprecedented detail and has allowed segregating NSCLC into discrete molecular subgroups, characterized by specific oncogenic drivers, such as epidermal growth factor receptor (EGFR), BRAF, KRAS, epidermal growth factor receptor 2 (HER2) mutations, MET amplification and anaplastic lymphoma kinase gene rearrangements (ALK).^2, 3^ Consequently, the understanding of NSCLC biology has brought two new classes of targeted agents into the clinical setting: EGFR tyrosine kinase inhibitors (TKIs) and ALK inhibitors.^4, 5^ In particular, clinical trials have shown that NSCLC patients whose tumors harbor sensitizing EGFR mutations significantly benefit from the upfront use of an EGFR TKI, rather than conventional chemotherapy.^6, 7, 8, 9, 10, 11^ Although licensed for clinical use in chemotherapy-pretreated patients, regardless of EGFR mutational status, the EGFR TKI erlotinib has limited efficacy when compared with standard chemotherapy in patients with WT-EGFR NSCLC.^12, 13, 14^

However, a fraction of patients on erlotinib treatment may achieve clinically significant objective responses and prolonged disease control, despite the lack of detectable EGFR mutations.^15^ Nevertheless, no biomarker investigated so far was felt sufficiently robust to select for the use of erlotinib in the maintenance or refractory setting.^16^ Thus, it would be crucial to identify molecular predictors of TKI sensitivity in EGFR wild-type (WT) tumors in order to prospectively select the subgroup of patients who may benefit from erlotinib therapy. Moreover, EGFR TKIs have also shown a modest therapeutic effect in lung squamous cell carcinoma (SCC), where EGFR mutations are very rare and patients have limited therapeutic options in the maintenance and relapsed settings.^16, 17, 18, 19, 20^

Even more importantly, in order to obtain meaningful clinical responses it is crucial to effectively target the population of cells that are able to escape treatment and maintain the growth of a resistant tumor.^21^ Cancer stem cells (CSCs) have been in fact identified within most solid tumors, including lung tumors, and are associated with increased resistance to therapies.^22, 23, 24, 25, 26, 27, 28, 29, 30^ Thus, the efficacy of innovative therapeutic strategies should be validated against these more aggressive, tumor-maintaining cells.^23, 27, 31^ Importantly, TKI response has never been determined at the level of the tumor-maintaining CSCs. Thus, we investigated erlotinib response of EGFR mutation-negative lung cancer stem cells (LCSCs) and LCSC-based xenografts with the attempt to evaluate their sensitivity to the drug and correlate it with their molecular pattern in order to identify potential biomarkers predictive of erlotinib response in a WT-EGFR context at the CSC level.

## Results

### Validation of LCSCs and response to EGFR TKI

**Figure 1:**
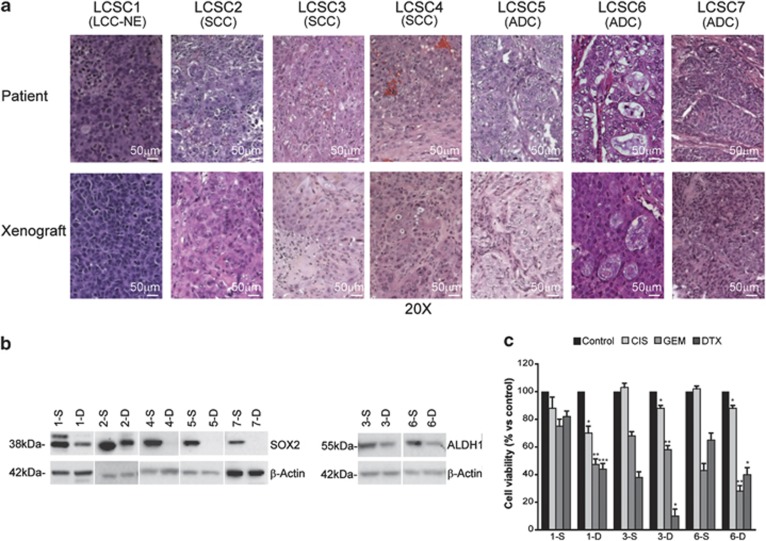
(**a**) LCSCs generated subcutaneous patient-like xenografts in NOG mice. Hematoxylin–eosin-stained sections of patient tumors (left panels) and mouse xenografts (right panels) obtained with the indicated LCSCs. Magnification is × 20 and scale bar corresponds to 50 *μ*m. LCC-NE, large-cell neuroendocrine carcinoma; SCC, squamous cell carcinoma; ADC, adenocarcinoma. (**b**) The *in vitro* differentiated LCSCs show decreased CSC-related gene expression. Immunoblot for SOX2 or ALDH1 in the indicated LCSCs (-S) or their *in vitro* differentiated progeny (-D). (**c**) The *in* vitro differentiated LCSCs gain chemosensitivity. Control or chemo-treated LCSCs and their corresponding *in vitro* differentiated progeny were left untreated (control) or exposed to cisplatin (CIS), gemcitabine (GEM) or docetaxel (DTX). Cell viability was measured after 48 h. Mean±S.D. of three independent experiments is shown. *P<0.05; **P<0.01; ***P<0.001

**Figure 2:**
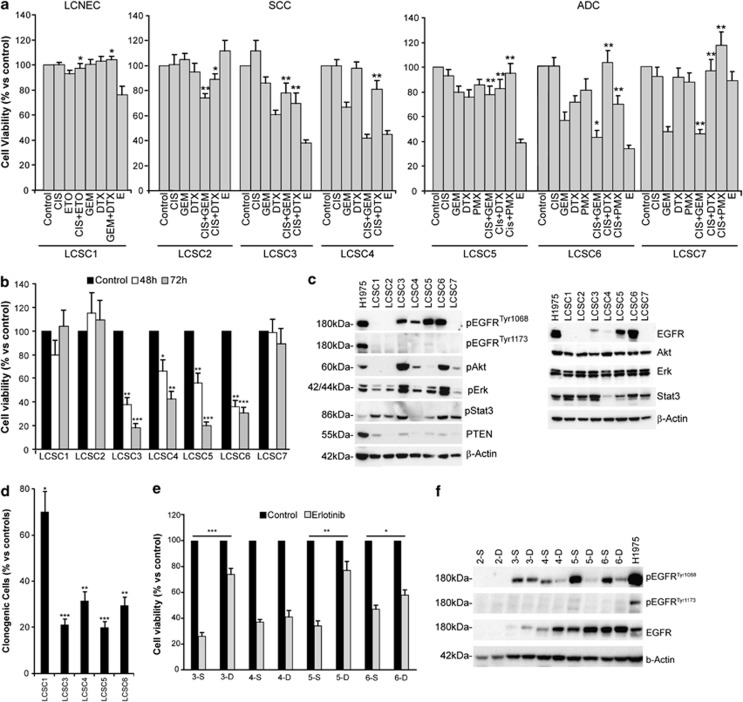
Cytotoxic activity of chemotherapy or erlotinib and EGFR pathway activation in LCSCs. (**a**) LCSCs were exposed to the indicated drugs and cell viability evaluated after 48 h and indicated as percentage *versus* control cells. (**b**) Time course of erlotinib-induced cytotoxicity. Cell viability was evaluated by CellTiter-Glo after 48 and 72 h of erlotinib exposure. (**c**) Immunoblot analysis of the indicated components of the EGFR pathways. (**d**) Long-term effects of erlotinib on LCSCs. Percentage of clonogenic cells in soft agar assay of erlotinib-treated *versus* control is indicated for each LCSC analyzed. (**e**) Cytotoxic activity of erlotinib in LCSCs (-S) and corresponding differentiated cells (-D) of each sample as indicated. Cells were exposed to erlotinib for 3 days and cell viability evaluated by CellTiter-Glo. (**f**) Immunoblot comparison of EGFR expression and activation in LCSCs (-S) and their *in vitro* differentiated counterparts (-D). Mean±S.D. of three independent experiments is always shown. *P<0.05; **P<0.01; ***P<0.001

LCSCs from WT-EGFR NSCLC patients with SCC (*n*=3), adenocarcinoma (ADC, *n*=3) and large-cell neuroendocrine carcinoma (LCNEC, *n*=1; Table 1a) were isolated as tumor spheres in serum-free culture conditions that enrich cultures for undifferentiated tumor cells endowed with stem cell properties of long-term proliferation capacity, increased clonogenic potential, differentiation ability, chemoresistance, increased tumorigenicity and ability to generate xenografts that mimic the tumor of origin, as we previously reported^24, 32, 33^ and have shown in Figure 1. Functional response of LCSC to erlotinib over 2-day *in vitro* exposure was then assessed in comparison with standard chemotherapy: cisplatin, gemcitabine, pemetrexed for ADC-derived LCSC, cisplatin, gemcitabine, docetaxel for SCC-derived LCSC and cisplatin, etoposide, gemcitabine or docetaxel for LCNEC-derived LCSCs. Four out of seven LCSC cell lines (SCC: LCSC3 and LCSC4; ADC: LCSC5 and LCSC6) were strikingly sensitive to erlotinib, with >50% reduction in cell viability; in these cells, erlotinib was as effective or more effective than cisplatin-based chemotherapy doublets (Figure 2a). Massive cell death was observed in erlotinib-sensitive LCSCs after longer (3 days) drug exposure (Figure 2b). Among other clinically relevant TKI inhibitors, cetuximab was essentially devoid of significant antitumor activity against LCSCs, whereas gefitinib displayed a substantial cytotoxic activity against the same erlotinib-sensitive LCSCs (Supplementary Figure 1A). Thus, sensitivity of LCSCs toward EGFR inhibition is not limited to erlotinib, but may be a general response to small=molecule EGFR inhibitors.

### Molecular characterization of LCSCs

EGFR, HER2, KRAS, PTEN and PI3K were sequenced for cancer-associated mutations (Table 1a, Supplementary Table 1 and Supplementary Information); in addition, HER2 and EGFR copy numbers or EML4-ALK (echinoderm microtubule-associated protein-like 4–anaplastic lymphoma kinase) rearrangement were evaluated by FISH (Table 1b and Supplementary Information). The entire EGFR gene sequencing was performed to evaluate the possible occurrence of EGFR mutations outside the clinically relevant regions (exons 18 through 21). No mutations in the EGFR, PTEN and PIK3CA genes or EML4-ALK fusions were found in the panel of LCSC lines analyzed (Table 1a); LCSCs 4 and 6 (SCC and ADC, respectively) displayed KRAS G12C (Table 1a,Supplementary Table 2 and Supplementary Information). However, EGFR gene copy number was increased in 5 out of 7 LCSCs and frankly amplified in 4 (Table 1b); the HER2 gene was frankly amplified in two SCC-derived LCSC cell lines (Table 1b).

### EGFR^tyr1068^ is associated with erlotinib sensitivity in EGFR-WT LCSCs

Partial correlation among erlotinib response of LCSCs and EGFR amplification was found. As expected, most LCSC lines with amplified EGFR were sensitive to erlotinib; however, LCSC1 displayed amplified EGFR and Erlotinib resistance, whereas LCSC6 displayed nonamplified EGFR and erlotinib sensitivity (Table 1b and Figure 2b). In the absence of EGFR mutations, we next evaluated EGFR protein expression and phosphorylation status in LCSCs. Strikingly, erlotinib-sensitive LCSCs displayed variable EGFR protein overexpression and highly consistent phosphorylation of the tyrosine 1068 (EGFR^tyr1068^) residue, as opposed to resistant LCSCs (Figure 2c). Conversely, tyrosine 1173 phosphorylation (EGFR^tyr1173^) was barely detectable (Figure 2c), as was phosphorylation of other EGFR sites including tyr1045 or tyr845 (not shown). We found broadly activated Akt, Erk or Stat3 pathways downstream of EGFR in both erlotinib-sensitive and -resistant LCSCs, without a discernible pattern (Figure 2c). Most LCSCs displaying high levels of EGFR expression and activation (LCSCs 3, 4, 5) harbored increased copies of EGFR gene (>8), suggesting that increased EGFR gene copies may contribute to overexpression and consequent activation of the receptor (Table 1). However, in LCSC6, EGFR was highly expressed and phosphorylated in the absence of increased gene copies, suggesting that other mechanisms may contribute to the activation of EGFR in this context (Figure 2c and Table 1b). Moreover, LCSC1 displayed EGFR amplification in the absence of EGFR activation or sensitivity. These results indicate that EGFR amplification does not always correlate with EGFR activation or erlotinib response in LCSCs. Overall, these data suggest that EGFR^tyr1068^ may represent a putative additional biomarker for EGFR TKI sensitivity in LCSCs.

### Erlotinib preferentially kills WT EGFR^tyr1068^ LCSCs compared with their differentiated progeny

We evaluated the long-term effect of erlotinib on LCSCs in colony formation assay. Erlotinib treatment dramatically reduced the ability of LCSCs with activated EGFR to generate colonies in soft agar assay, demonstrating long-term efficacy of the drug and its ability to target the clonogenic cells (Figure 2d). We next compared LCSC response with erlotinib and EGFR activation level with that of their *in vitro* differentiated counterpart (Figures 2e and f). In erlotinib-sensitive LCSCs, EGFR^tyr1068^ expression was more prominent in stem *versus in vitro* differentiated cell populations (Figure 2f). Consistent with the proposed role of EGFR^tyr1068^ in marking an EGFR-addicted functional state, erlotinib-induced cytotoxicity against LCSCs was even more marked than that observed in the differentiated cells (Figure 2e). *In vitro* differentiation was substantiated by reduction of embryonic gene expression (ALDH1, SOX2) and gain of chemosensitivity, as shown in Figures 1b and c and our previous data.^32, 33^ In line with these results, drug treatment of the LCSC population determined a reduction of the stemness-related aldehyde dehydrogenase (ALDH) expression. Based on the assumption that tumor spheres are highly enriched in CSCs although containing cells with lower degree of stemness, these results confirm that erlotinib preferentially killed the more undifferentiated cells within the LCSC culture (Supplementary Figure 1B).

### EGFR^tyr1068^ association with EGFR-sensitizing mutations in lung cancer cell lines and patient tumors

Based on the results reported above, we extended the study to a panel of commercial lung cancer cell lines. All of the cell lines with known EGFR-activating mutations (HCC827, H1975 and H1650) and the EGFR-WT Calu3 cell line displayed prominent EGFR^tyr1068^ phosphorylation and were sensitive (<30% reduction of cell viability) to erlotinib (Supplementary Figures 2A–C). Conversely, EGFR^tyr1173^ was not associated with EGFR mutational status or erlotinib sensitivity (Supplementary Figures 2A–C).

We also analyzed the expression of EGFR^tyr1068^ and EGFR^tyr1173^ in a series of 91 lung cancer specimens, with (*n*=39) or without (*n*=52) EGFR-sensitizing mutations (Table 2). In this series, EGFR^tyr1068^ was preferentially expressed in EGFR-mutant samples (score 2+/3+ in 64% of EGFR-mut cases *versus* 38% of EGFR-WT cases, respectively; *P*=0.03), whereas EGFR^tyr1173^ similarly distributed in EGFR-mut and EGFR-WT samples (score 2+/3+ in 38% and 37% of cases, respectively; *P*=0.97) (Table 2c). Overall, these data support the idea that EGFR^tyr1068^, as opposed to EGFR^tyr1173^, marks an EGFR activation state driven by activating EGFR gene mutations; when constitutively present in an EGFR-WT genetic context, such activation state may still portend sensitivity to EGFR TKI even in the absence of EGFR gene mutations.

### Erlotinib treatment-induced EGFR pathway downregulation and apoptosis in LCSCs with activated EGFR

**Figure 3:**
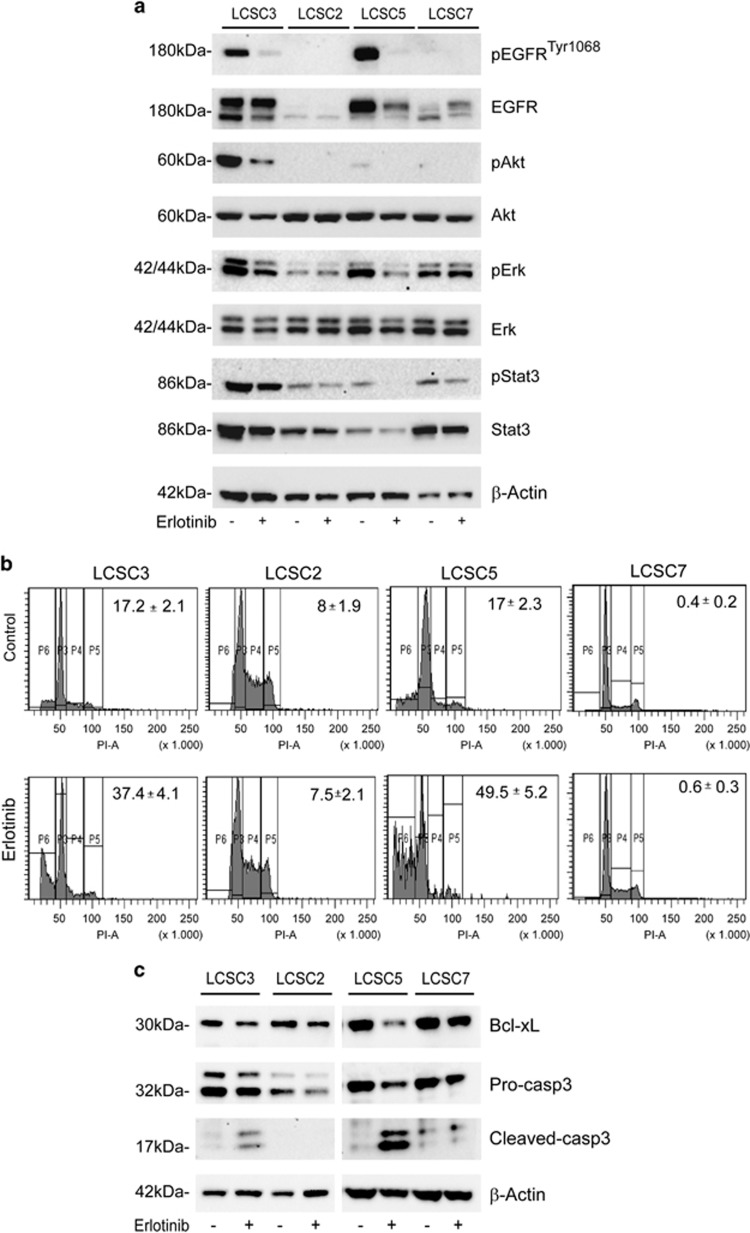
Erlotinib-induced EGFR pathway downmodulation and cell death in sensitive LCSCs. (**a**) Immunoblot analysis of the indicated EGFR pathway components in control or 2-day erlotinib-treated sensitive (LCSC3 and LCSC5) or resistant (LCSC2 and LCSC7) LCSCs. Flow cytometric quantification of propidium iodide-stained apoptotic cells (**b**) and immunoblot analysis of Bcl-xL and caspase-3 cleavage (**c**) in the same cells as in (**a**) exposed to erlotinib for 3 days. (**b**) Percentage of subdiploid/apoptotic cells is indicated±S.D.

At the molecular level, erlotinib induced EGFR pathway downregulation in all LCSCs bearing activated receptor. EGFR^tyr1068^ and all downstream mediators of EGFR signaling analyzed, p-STAT3, p-AKT and p-ERK, although expressed at variable extent in the different samples, displayed consistent decrease in sensitive LCSCs after erlotinib exposure (Figure 3a). These results suggest that decreased activation of all pathways contributed to mediate erlotinib-induced toxicity in LCSCs, and this is in agreement with previously reported data in lung cancer cell lines.^34, 35^ Analysis of DNA content by propidium iodide staining and flow cytometry analysis of control and erlotinib-treated cells revealed the appearance of the sub-diploid DNA peak, hallmark of apoptosis, specifically in erlotinib-sensitive treated samples. A 20% (LCSC3) or 32.5% (LCSC5) increase in the apoptotic cells fraction was measured in sensitive LCSCs, whereas a negligible variation was detected in resistant LCSC2 and LCSC7 after erlotinib exposure (Figure 3b). Decrease of the anti-apoptotic protein Bcl-xL (B-cell lymphoma-extra large) and cleavage of caspase-3 detected by immunoblotting further confirmed that erlotinib cytotoxicity occurred through apoptosis induction in responsive cells (Figure 3c).

### Specificity of erlotinib antitumor activity in LCSC-generated ADC xenografts with activated EGFR

**Figure 4:**
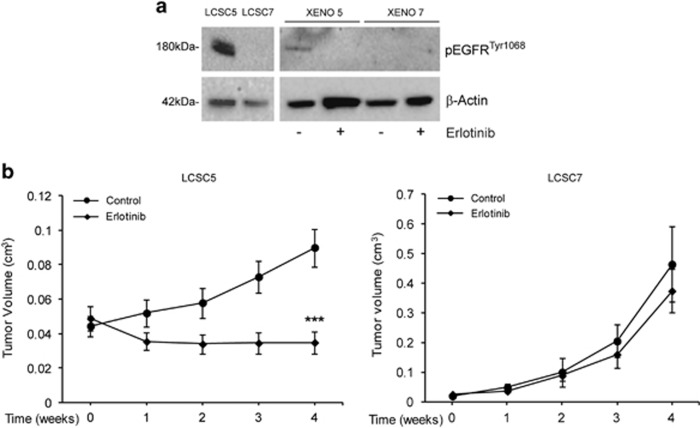
EGFR activation and Erlotinib antitumor activity in LCSC-derived ADC xenografts. (**a**) Immunoblot analysis of EGFR^tyr1068^ in sensitive (LCSC5) or resistant (LCSC7) LCSCs and in the corresponding xenografts untreated (−) or treated (+) with erlotinib. (**b**) Growth curves of the same control or erlotinib-treated xenografts as in (**a**). Mean±S.D. of three independent experiments is shown. ***P<0.001

In order to evaluate the antitumor activity of erlotinib *in vivo*, LCSCs of ADC subtype either bearing or lacking EGFR^tyr1068^ were used to generate subcutaneous tumors and erlotinib was administered by oral gavage, following clinically used dosages and regimens, to ensure systemic drug concentrations comparable to those reached in clinical context. EGFR^tyr1068^ could be detected in CSC-generated xenografts, its expression correlated with that found in the corresponding LCSCs they were generated from and decreased following Erlotinib treatment *in vivo*, indicating that the erlotinib concentration achieved within the tumor was sufficient to inhibit EGFR activity (Figure 4a). The levels of EGFR^tyr1068^ were reduced in xenografts compared with the LCSCs, confirming the reduced EGFR activation also observed following differentiation *in vivo* (Figures 2e and 4a). Thus, we assumed that LCSC-generated xenografts constituted a highly reliable preclinical system suitable to be used to investigate erlotinib activity *in vivo*. Tumor growth was significantly inhibited by erlotinib exclusively in xenografts derived from EGFR-activated LCSCs, supporting the results *in vitro* (Figure 4b). Thus, erlotinib exerted a marked antitumor activity *in vivo* exclusively in EGFR^tyr1068^-positive CSC-generated xenografts.

### Erlotinib antitumor activity in xenografts generated by ADC and SCC LCSCs with activated EGFR is superior than chemotherapy

Chemotherapy is currently the standard of care for ADC lung cancer patients in the absence of EGFR or other targetable molecular alterations (EML4-ALK), with erlotinib approved only for EGFR-mutated ADC patients in first-line treatment. As we found that the lung ADC CSC-derived xenografts with activated WT-EGFR are highly sensitive to erlotinib *in vivo*, we compared erlotinib with chemotherapy antitumor activity in this preclinical *in vivo* model in view of a possible therapeutic use of erlotinib for subgroups of mutation-negative patients with activated receptor.

**Figure 5:**
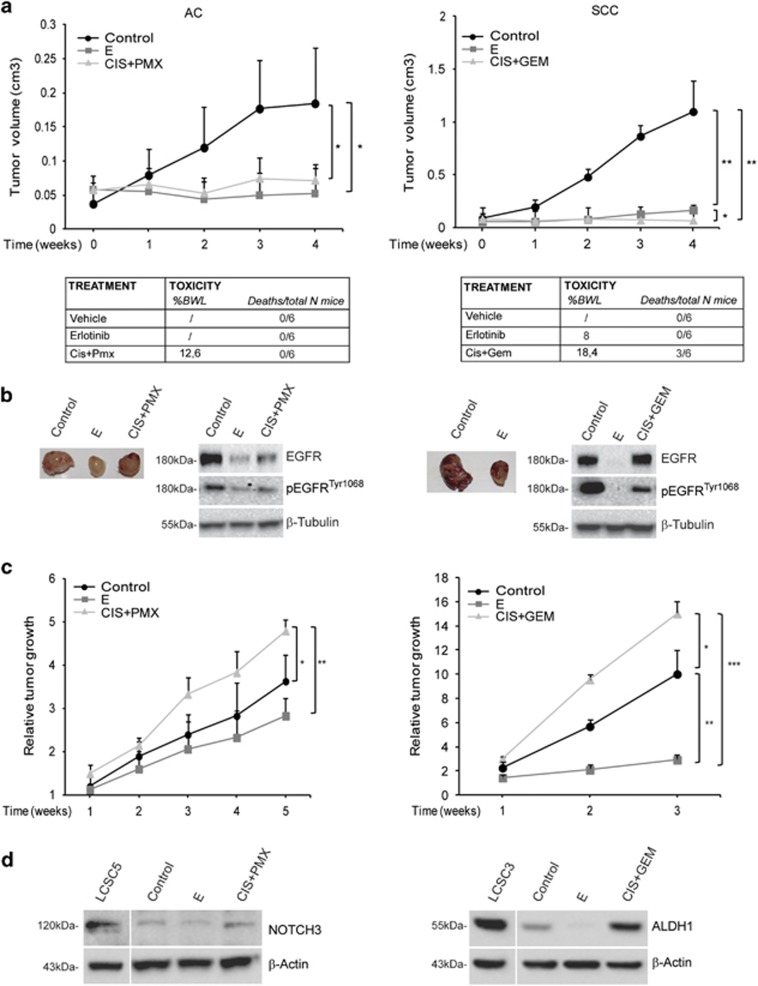
The *in vivo* antitumor activity of Erlotinib or chemotherapy in LCSC-generated adenocarcinoma (ADC) or squamous cell carcinoma (SCC) xenografts. (**a**, upper panels) Growth curves of LCSC-derived xenografts in control mice or mice treated with erlotinib, cisplatin/pemetrexed combination (Cis+Pem) or cisplatin/gemcitabine combination (Cis+Gem), as indicated (). Mean±S.D. of three independent experiments is shown. *P<0.05; **P<0.01. (**a**, lower panels) Table of drug-induced systemic toxicity in the three groups of mice indicated as percentage of body weight loss (BWL) or number of deaths/total number of mice. (**b**) Images of tumors at the end of each treatment and immunoblot analysis of EGFR/pEGFR^tyr1068^ in cells obtained from control or treated tumors. (**c**) Relative tumor growth of control or pretreated tumors after treatment interruption. Relative tumor growth is indicated as ratio of tumor volume at the indicated week after drug suspension *versus* volume at the last day of treatment. *P<0.05; **P<0.01; ***P<0.001. (**d**) Immunoblot analysis of the indicated CSC-related proteins in control or treated xenografts in comparison with their corresponding LCSCs

Moreover, not only ADC but also SCC type of LCSCs were highly sensitive to erlotinib *in vitro* (Figure 2a). Therefore, we investigated the *in vivo* activity of erlotinib in comparison with that of chemotherapeutic doublets (cisplatin/pemetrexed for ADC and cisplatin/gemcitabine for SCC). During drug treatment, growth was impaired to a similar extent by erlotinib and chemotherapy in both xenograft models derived from EGFR^tyr1068^-positive LCSCs (Figure 5a). Erlotinib was highly tolerated at the used dosages as it did not determine adverse effects in treated mice, except for a light body weight loss. In contrast, chemotherapy was highly toxic determining marked weight loss in all mice and toxic deaths in a few mice (Figure 5a, bottom panels). Tumor growth inhibition was clearly visible in excised xenografts and was associated with decrease of EGFR^tyr1068^ in treated tumors (Figure 5b). Following treatment interruption, chemotherapy pretreated tumors started to overgrow to a rate much higher than control xenografts, as expected for tumors whose more aggressive tumorigenic cells are spared by chemotherapy and in line with the clinical behavior of chemo-relapsed patient tumors (Figure 5c). In line with their increased growth rate, tumor cells obtained from chemo-treated tumors displayed an augmented expression of the CSC-related genes ALDH1 and NOTCH3 compatible with a higher CSC content and supporting the evidence of a more aggressive phenotype of tumors following chemotherapy (Figure 5d). Although highly expressed in the ADC LCSCs, the CSC-related gene SOX2 was undetectable in corresponding xenograft, suggesting that its expression was drastically abolished following differentiation *in vivo*, confirming the results observed *in vitro* (Figures 1b and 5d). Thus, as the expression of SOX2 was likely restricted to the small CSC subpopulation, the limited protein amount in xenografts did not allow its detection.

In contrast, reduced expression of CSC-related genes was observed in tumors following erlotinib therapy, consistent with a lower CSC content of erlotinib-treated tumors, in line with their decreased growth rate and supporting the assumption of a preferential activity of erlotinib against the tumor-maintaining cells also *in vivo* (Figure 4d). Finally, in agreement with *in vitro* results, we found that erlotinib antitumor activity also occurred through apoptosis induction *in vivo*, as demonstrated by the reduction of pro-caspase 3 and the antiapoptotic protein Bcl-XL in cell lysates derived from erlotinib-treated xenografts in comparison with controls (Supplementary Figure 3).

## Discussion

EGFR-activating mutations have been widely proven to be associated with increased patient response to anti-EGFR therapies, thus becoming the indication for erlotinib treatment in NSCLC patients of ADC subtype.^7^ Although secondary resistance invariably occurs because of multiple mechanisms, erlotinib therapy has proved superior to chemotherapy in the subgroup of EGFR-mut patients, in term of increased response rate and reduced toxicity.^7, 8, 9, 10, 11, 36^ However, EGFR mutations occur with low frequency (∼30%) in ADC subtype and are virtually absent in SCC in the Caucasian population, and thus most patients are currently excluded from erlotinib therapeutic option either as first-line therapy or in recurrence/maintenance clinical protocols, following chemotherapeutic regimen failure.^37^ Consequently, treatment of EGFR mutation-negative patients represents a relevant issue in NSCLC management, considering the scarcity of therapeutic opportunities available, particularly for SCC patients. Importantly, a fraction of NSCLC patients have displayed partial response to erlotinib despite lack of EGFR mutations and the identification of molecular biomarkers of response would be of great clinical value to prospectively select those EGFR-WT patients who are likely to benefit from erlotinib therapy.^16, 17, 18, 19^

As it has been widely accepted that most solid tumors, including lung tumors, arise and are maintained by CSCs, and that chemotherapy-spared CSCs are responsible for tumor recurrence, it is clear that biomarkers predicting erlotinib response in EGFR-WT tumors have to be determined at the CSC level. In this study, we investigated erlotinib response of LCSCs with WT-EGFR and identified EGFR phosphorylation at tyrosine 1068 (EGFR^tyr1068^) as a powerful biomarker associated with strong erlotinib sensitivity both *in vitro* and in preclinical CSC-generated subcutaneous xenografts. Importantly, in contrast to the preferential activity of chemotherapy against differentiated cells, erlotinib cytotoxicity was even more marked against the LCSCs than against their differentiated counterpart both *in vitro* and in xenografts, acquiring a great value as a CSC-directed therapeutic drug in the context of WT-EGFR lung cancers. Although displaying similar ability to inhibit tumor growth during treatment, erlotinib proved superior to chemotherapy in terms of tolerability and reduced tumor aggressiveness following treatment interruption. Among the two most relevant sites of phosphorylation, tyr1068 and tyr1173, only tyr1068 was phosphorylated in LCSCs, thus increasing the relevance of this site of phosphorylation as a biomarker associated with the tumor-maintaining cells, and thus representing a potential therapeutic target for a long-lasting therapeutic inhibition.

Finally, besides its invariable association with erlotinib sensitivity in EGFR-WT LCSCs, EGFR^tyr1068^ was associated with EGFR-sensitizing mutations in cell lines and patient tumors, with relevant diagnostic and therapeutic implication.

Correlation of EGFR^tyr1068^ expression and EGFR inhibitor sensitivity was also found in commercial lung cancer cell lines, where EGFR^tyr1068^ was strictly associated with sensitivity regardless of EGFR mutational status. In contrast, EGFR^tyr1173^ was not correlated with sensitivity or mutation (Supplementary Figure 2). Among the erlotinib-responsive lung cancer cell lines, we found that H1975 and H1650 cells were endowed with a lower degree of sensitivity to the drug compared with HCC827, in agreement with the previously demonstrated modulation of erlotinib response by lack of functional PTEN (H1650) or by the concomitant presence of the T790M EGFR resistance mutation (H1975) in these cells.^38, 39, 40^ This result suggested that an intact AKT inhibitory signaling (PTEN) is required for erlotinib-induced cytotoxicity of lung cancer cells with mutated EGFR. Similarly, it is likely that the genetic background of EGFR-activated lung cancer cells may also modulate the extent of erlotinib sensitivity in EGFR-WT cells. We found that LCSC4 and LCSC6 were highly sensitive to erlotinib despite the presence of mutated KRAS (Kirsten rat sarcoma), indicating that KRAS mutation does not affect erlotinib-induced cytotoxicity in LCSCs with WT/activated receptor (Table 1a and Figure 2). This result is in agreement with previous reports showing that although KRAS mutation is a general negative prognostic factor, lung cancer patient response to erlotinib is independent of KRAS status.^41, 42^ Erlotinib dose used *in vitro* belongs to the higher ranges of drug concentrations used in other studies with EGFR-mutated cell lines that may display higher responsiveness to EGFR inhibition, particularly at lower doses. Nevertheless, at the used concentration, erlotinib displayed strong activity against the LCSCs with EGFR^tyr1068^ *in vitro*. Moreover, doses and schedules used *in vivo* were compatible with those used in clinical setting, and under these conditions treatment was endowed with high antitumor activity specifically against the WT/activated EGFR tumors and highly tolerated, excluding the possibility that the observed *in vitro* activity of erlotinib could result from drug overdosage, not achievable *in vivo*.

We found that erlotinib sensitivity of EGFR-WT LCSCs was even higher when compared with their differentiated counterpart, in line with their higher level of receptor activation (Figure 2e and f). It has been reported that in EGFR-mutated lung cancer cell lines EGFR blockade may enrich for lung cancer stem-like cells that resist the treatment.^43^ The apparent discrepancy may lay in the type of EGFR activation occurring in EGFR-mutated or -WT cells. In EGFR-WT context we showed that the activation of EGFR is modulated in the different cell compartments, with the extent of EGFR activation markedly higher in the CSCs compared with differentiated cells, substantiating the latter reduced sensitivity to erlotinib. In contrast, gene mutation-dependent EGFR activation, being constitutive, might not vary in different cell compartments determining a similar basal potential of response in CSCs and differentiated cells. In spite of this, EGFR activation being equal, the response to erlotinib may be reduced in the CSCs because of their intrinsic pro-survival CSC properties (drug extrusion ability, expression of resistance genes, increased ability to escape cell death, etc.). Based on these assumptions, it is reasonable that erlotinib treatment could spare LCSCs in the mutated-EGFR context, whereas it may preferentially target LCSCs in EGFR mutation-negative tumors.

Erlotinib also displayed a stricking antitumor efficacy in EGFR^tyr1068^-positive LCSC-generated xenografts. Erlotinib and chemotherapy inhibited tumor growth rate to a similar extent during drug administration, although chemotherapy determined a massive systemic toxicity in a fraction of animals, whereas erlotinib was highly tolerable (Figure 4a). However, after treatment interruption, chemotherapy-exposed tumors displayed increased aggressiveness, as expected for tumors whose more malignant tumorigenic cells are spared by chemotherapy and in line with the behavior of chemo-treated clinical tumors. In contrast, growth rate of erlotinib-pretreated tumors was even inferior than control tumors, and expression of CSC-related genes was decreased, consistent with a minor CSC content of these tumors and supporting the *in vitro* evidence of a preferential CSC-directed cytotoxicity by erlotinib. Thus, we found that the antitumor efficacy exerted by erlotinib in xenografts generated by EGFR^tyr1068^-positive LCSCs was superior to that obtained with chemotherapy in terms of long-term efficacy and tolerability and, importantly, it occurred both in ADC and SCC lung cancer subtypes, with relevant clinical implication as SCC patients have very limited therapeutic options besides chemotherapy, at present.

Moreover, we found that EGFR^tyr1068^, and not EGFR^tyr1173^, was associated with EGFR-sensitizing mutations in cell lines and patient tumors. Thus, EGFR^Tyr1068^ was associated with lung cancer cells and tumors bearing EGFR-sensitizing mutations or with lung cancer cells and LCSCs that were sensitive to erlotinib treatment despite lack of EGFR mutation. In this hypothesis, EGFR^tyr1068^ immunohistochemistry could represent a surrogate tool besides EGFR sequencing analysis to predict potential erlotinib sensitivity, applicable also among mutation-negative patients and CSC based. However, future studies of patient outcome will contribute to determine whether the level of EGFR^tyr1068^ detected in patient tumors would identify mutation-negative tumors with activated receptor, more likely responsive to erlotinib.

In conclusion, our studies add a potential further level of molecular determinants for erlotinib sensitivity besides gene mutation, amplification or increased copy number that have been considered for clinical studies so far but do not always take for granted EGFR activation or erlotinib response.

## Materials and Methods

### Isolation and culture of lung cancer stem cells

Tumor samples were obtained in accordance with consent procedures approved by the internal review board of Department of Laboratory Medicine and Pathology, Sant'Andrea Hospital, University La Sapienza, Rome. Tumor tissue dissociation and procedures for medium preparation and expansion of LCSC *in vitro* were performed as we previously described.^24^ Briefly, tissue dissociation of surgical specimens was carried out by enzymatic digestion (20 *μ*g/ml collagenase II, 20 *μ*g/ml DNAse I, Gibco-Invitrogen, Carlsbad, CA, USA) for 2 h at 37 °C. Recovered cells were cultured in serum-free medium containing 50 *μ*g/ml insulin, 100 *μ*g/ml apo-transferrin, 10 *μ*g/ml putrescine, 0.03 *μ*M sodium selenite, 2 *μ*M progesterone, 0.6% glucose, 5 mM hepes, 0.1% sodium bicarbonate, 0.4% BSA, glutamine and antibiotics, dissolved in DMEM-F12 medium (Gibco-Invitrogen) and supplemented with 20 ng/ml EGF and 10 ng/ml b-FGF. Flasks nontreated for tissue culture were used in order to reduce cell adherence and support growth as undifferentiated tumor-spheres. Medium was replaced or supplemented with fresh growth factors twice a week until cells started to grow, forming floating aggregates. Cultures were expanded by mechanical dissociation of spheres, followed by replating of both single cells and residual small aggregates in complete fresh medium. In order to obtain differentiation of lung cancer sphere-forming cells, stem cell medium was replaced with bronchial epithelial cell growth medium (BEGM, Lonza, East Rutherford, NJ, USA) in tissue culture-treated flasks to allow cell attachment and differentiation. Loss of stem cell markers and functions as well as gain of chemosensitivity were considered for LCSC validation (Figure 1a and our previous results^24, 32, 33^).

### Cell line culture and drug treatments and cell viability assay

Lung cancer cell lines H1299, H299, Calu1, H460, H1975, H1650, Calu3 and HCC827 were obtained from ATCC (Manassas, VA, USA) and grown in 10% fetal bovine serum containing complete RPMI medium (Gibco-Invitrogen). The cell lines were obtained directly from the ATCC that performs cell line characterizations or authentication by the short tandem repeat profiling and passaged in our laboratory for >6 months after receipt. For drug treatments and cell viability assays, 3000 cells were plated in triplicate in 96-well plate and left untreated or exposed for 3 days to the following drugs before CellTiter-Glo evaluation (Promega, Madison, WI, USA): 10 *μ*M erlotinib, 10 *μ*M gefitinib and 100 *μ*g/ml cetuximab.

### Chemotherapy and erlotinib treatment

A total of 3000 cells obtained from sphere dissociation were plated in 96-well flat-bottom plates. Chemotherapeutic agents (Sigma, St. Louis, MO, USA) were added at the following final concentrations: cisplatin 1 *μ*g/ml, gemcitabine 50 *μ*M, docetaxel 0.5 *μ*g/ml, pemetrexed 100 *μ*g/ml and etoposide 5 *μ*g/ml; EGFR inhibitor erlotinib (Genentech, South San Francisco, CA, USA) at the final concentration of 10 *μ*M. Cell viability was evaluated by both luminescent cell viability assay (CellTiter-Glo, Promega) and cell count by Trypan blue exclusion. For soft agar assay, 500 single cells were plated in the top agar layer in each well of a 24-well culture plate with 0.3% top agar layer and 0.4% bottom agar layer (SeaPlaque Agarose, Cambrex, NJ, USA). Cells were cultivated at 37  °C for 3 weeks and colonies from triplicate wells were stained with crystal violet (0.01% in 10% MetOH), visualized and counted under microscope.

### Western blot

For immunoblotting studies, 20 *μ*g of proteins from each sample were resolved on 4–12% polyacrylamide gel electrophoresis NuPAGE Bis-Tris (Invitrogen, Carlsbad, CA, USA) and transferred to nitrocellulose membranes. Rabbit polyclonal anti-Phospho-EGFR (Tyr1068), -Phospho-EGFR (tyr1173), -Phospho-Akt (Ser473) -Akt, -Caspase-3 (8G10) and -Phospho-Stat3 (Ser727) and mouse monoclonal anti-STAT3 were purchased from Cell Signaling (Beverly, MA, USA). Mouse monoclonal anti-PTEN was purchased from BD (Franklin Lake, NJ, USA), mouse monoclonal anti-Phospho-ERK clone (E-4), rabbit polyclonal anti-EGFR (1005), -Bcl xL (H5), -Erk and -Notch3 were purchased from Santa Cruz Biotechnology (Santa Cruz, CA, USA) and mouse monoclonal *β*-tubulin and *α*-actin were purchased from Sigma-Aldrich (St. Louis, MO, USA). Peroxidase-conjugated secondary antibodies were purchased from Amersham Pharmacia Biotech (Buckinghamshire, UK).

### Apoptosis assay

For apoptosis assay 1 × 10^5^ cells were washed with PBS and resuspended in Nicoletti buffer (0.1% sodium citrate, pH 7.4/0.1% Triton X) containing 100 *μ*g/ml propidium iodide and 200 *μ*g/ml RnaseA. After 2 h of incubation at 4 °C, samples were analyzed with FACSCAN (Beckton Dickinson).

### Xenograft generation and mice treatments

Cell suspensions of ADC and SCC CSC lines were mixed 1 : 1 with growth factor-reduced Matrigel (BD) and injected subcutaneously in the flanks of 4-week-old female NSG (NOD/SCID nonobese diabetic/severe combined immunodeficiency gamma chain deficient) mice (Charles River, Wilmington, MA, USA). For drug treatment, when tumors reached a mean of 0.5 cm diameter, mice were treated with vehicles, erlotinib (100 mg/kg/5 days on and 2 days off/gavage) freshly dissolved in 0.5% hydrossimethylcellulose/0.1% Tween-80, chemotherapeutic agent combinations as cisplatin (3 mg/kg /biweekly/intraperitoneally (IP)) + pemetrexed (200 mg/kg /biweekly/IP) or cisplatin (3 mg/kg /biweekly/IP) + gemcitabine (60 mg/kg /biweekly/IP). Tumors were measured once a week for the 4 weeks using a caliper, and mice were monitored for signs of drug-induced toxicity and weighed regularly. At the end of treatments, tumors were monitored for 3–5 weeks or collected and dissociated to obtain cell suspension as indicated above and previously reported.^24^

### Immunohistochemistry on tumor sections

EGFR expression was detected by IHC using EGFR PharmDX kit (Dako, Glostrup, Denmark) according to the manufacturer's instructions. Antigen retrieval was performed treating sections by proteinase K. Phospho-EGF Receptor (Tyr1173) (EGFR^tyr1173^) and Phospho-EGF Receptor (Tyr1068) (EGFR^tyr1068^) expression was detected by IHC using specific monoclonal antibodies (Cell Signaling). Antigen retrieval was performed at 96 °C using a 10 mM EDTA buffer, pH 8, for 40 min in a thermostatic bath. Diaminobenzidine (DAB) was used as chromogenic substrate. EGFR, p-EGFR^tyr1068^ and p-EGFR^tyr1173^ were interpreted according to the follow scoring criteria: negative, no reaction; 1+, 2+ or 3+, if neoplastic cells displayed a weak, moderate or strong plasmamembrane immunostaining, respectively.

### Statistical analysis

The statistical significance of the results was evaluated by ANOVA and Bonferroni post tests. All statistical tests were performed using GraphPad Prism v.4.0 for Windows (GraphPad Software, San Diego, CA, USA, www.graphpad.com) and the threshold for statistical significance was set at 0.05. *P*-values are displayed on the graphs using a single asterisk for significances ranging from 0.05 to 0.01, two asterisks for values between 0.01 and 0.001 and three asterisks for values below 0.001.

## Figures and Tables

**Table 1A:** Clinical staging and classification of lung cancer samples and mutational status of the lung cancer-associated gene in the corresponding LCSCs

	**Tumor type**	**KRAS**	**EGFR**	**EML4-ALK fusion**	**PTEN**	**PI3KCA**	**HER2**
LCSC1	LCC-NE (pT2pN2pMx-IIIA	WT	WT	No	WT	WT	WT
LCSC2	SCC (pT2pN2pMX(IIIA)-G2)	WT	WT	No	WT	WT	WT
LCSC3	SCC (pT3pN0pMx-IIB-G3)	WT	WT	No	WT	WT	WT
LCSC4	SCC (pT2N0-IB)	Mut G12C(ggt>tgt)	WT	No	WT	WT	WT
LCSC5	AC (pT2pN2pMx-IIIA-G2)	WT	WT	No	WT	WT	WT
LCSC6	AC (pT4pN1IIIA-G3)	Mut G12C(ggt>tgt)	WT	No	WT	WT	WT
LCSC7	AC (pT2a pN0 M1-G3)	WT	WT	No	WT	WT	WT

**Table 1B:** Descriptive tables of EGFR (upper table) and HER2 (lower table) FISH analysis in LCSCs

**CSC cell line**	**EGFR gene copy number (mean)**	**CHR 7 centromere copy number (mean)**	**Ratio EGFR/Chr7**	**EGFR gene status**	**No. of counted cells**
LCSC1	5.9	2.01	2.92	Amplification^a^	185
LCSC2	5.2	2.9	1.79	Gain^b^	138
LCSC3	10.3	3.71	2.77	Amplification^a^	253
LCSC4	8.5	2.65	3.2	Amplification^a^	180
LCSC5	9.2	3.26	2.82	Amplification^a^	178
LCSC6	2.24	1.68	1.34	NA^c^	123
LCSC7	2.26	1.98	1.14	NA^c^	100

**CSC cell line**	**HER2 gene copy number (mean)**	**CHR 17 centromere copy number (mean)**	**Ratio HER2/Chr17**	**HER2 gene status**	**No. of counted cells**
LCSC1	3	2	1.5	NA^c^	100
LCSC2	4	2	>2	Amplification^a^	120
LCSC3	>10	2	>2	Amplification^a^	115
LCSC4	3	2	1.5	NA^c^	80
LCSC5	2	2	1	NA^c^	100
LCSC6	3	2	1.5	NA^c^	60
LCSC7	2	3	0.6	NA^c^	100

a Amplification: ratio (EGFR/Chr7 and HER2/Chr17) >2

b Increased gene copy number and chr7 polysomy

c No amplification, no polysomy

**Table 2A:** Correlation between EGFR, pEGFR^tyr1068^ and pEGFR^tyr1173^ expression and EGFR mutational status in 91 NSCLC patient tumors. (a) Patient information and clinical–pathological characteristics of NSCLC tumors

**Patient and tumor information**	**No. of patients**	**Percentage (%)**
*Gender/age*		
Male	40	44%
Female	51	56%
Median age	60.6	

*Histotype*		
Adenocarcinoma	76	83%
Squamous carcinoma	9	10%
Other	6	7%

*Grading*		
G1	1	1%
G2	25	28%
G3	44	48%
Unknown	21	23%

*Stage*		
I	9	10%
II	4	4%
III	16	18%
IV	38	42%
Unknown	24	26%

*EGFR status*		
WT	52	57%
MUT	39	43%

**Table 2B:** Correlation between EGFR, pEGFR^tyr1068^ and pEGFR^tyr1173^ expression and EGFR mutational status in 91 NSCLC patient tumors. (b) Type of EGFR gene mutation in the 39 mutated NSCLC tumors

**EGFR mutation**		
***Exon 18***	***Exon 19***	***Exon 21***
1(G719X) 2.5%	20 (deletions) 51%	13 (L858R) 34%
	1 (P741S) 2.5%	1 (P848L) 2.5%
	1 (V742I) 2.5%	1 (A859T) 2.5%
		1 (G873E) 2.5%
TOT 2.5%	TOT 56%	TOT 41.5%

**Table 2C:** Relationship between negative and positive p-EGFR1068/1173 in EGFR mutated (mut) or nonmutated (WT) patient samples

	**p-EGFR**^**Tyr1068**^		**p-EGFR**^**Tyr1173**^	
	0/1+	2+/3+	0/1+	2+/3+
Mut	36%	64%	62%	38%
WT	62%	38%	63%	37%

Percentages of negative/weakly positive (0/1+) or positive (2+/3+) staining are indicated

## Abbreviations

EGFR: epidermal growth factor receptor
TKI: tyrosine kinase inhibitor
CSC: cancer stem cell
HER2: human epidermal growth factor receptor 2
EML4-ALK: echinoderm microtubule-associated protein-like 4–anaplastic lymphoma kinase
BRAF: B-Raf proto-oncogene serine/threonine kinase
KRAS: Kirsten rat sarcoma
LCSC: lung cancer stem cell
NSCLC: non-small-cell lung cancer
ADC: adenocarcinoma
SCC: squamous cell carcinoma
LCNEC: large-cell neuroendocrine carcinoma
Bcl-XL: B-cell lymphoma-extra large
ALDH: aldehyde dehydrogenase
NSG: NOD/SCID nonobese diabetic/severe combined immunodeficiency gamma chain deficient
WT: wild type
Mut: mutated
IP: intraperitoneal
EGFR^1068^: Tyr1068-phosphorylated epidermal growth factor receptor
EGFR^1173^: Tyr1173-phosphorylated epidermal growth factor receptor
