# Supplementary figures and images for: Tyr1068-phosphorylated epidermal growth factor receptor (EGFR) predicts cancer stem cell targeting by erlotinib in preclinical models of wild-type EGFR lung cancer
Source: Cell Death Dis. 2015 Aug 6;6(8):e1850–. doi: 10.1038/cddis.2015.217 (PMC4558509; doi:10.1038/cddis.2015.217)

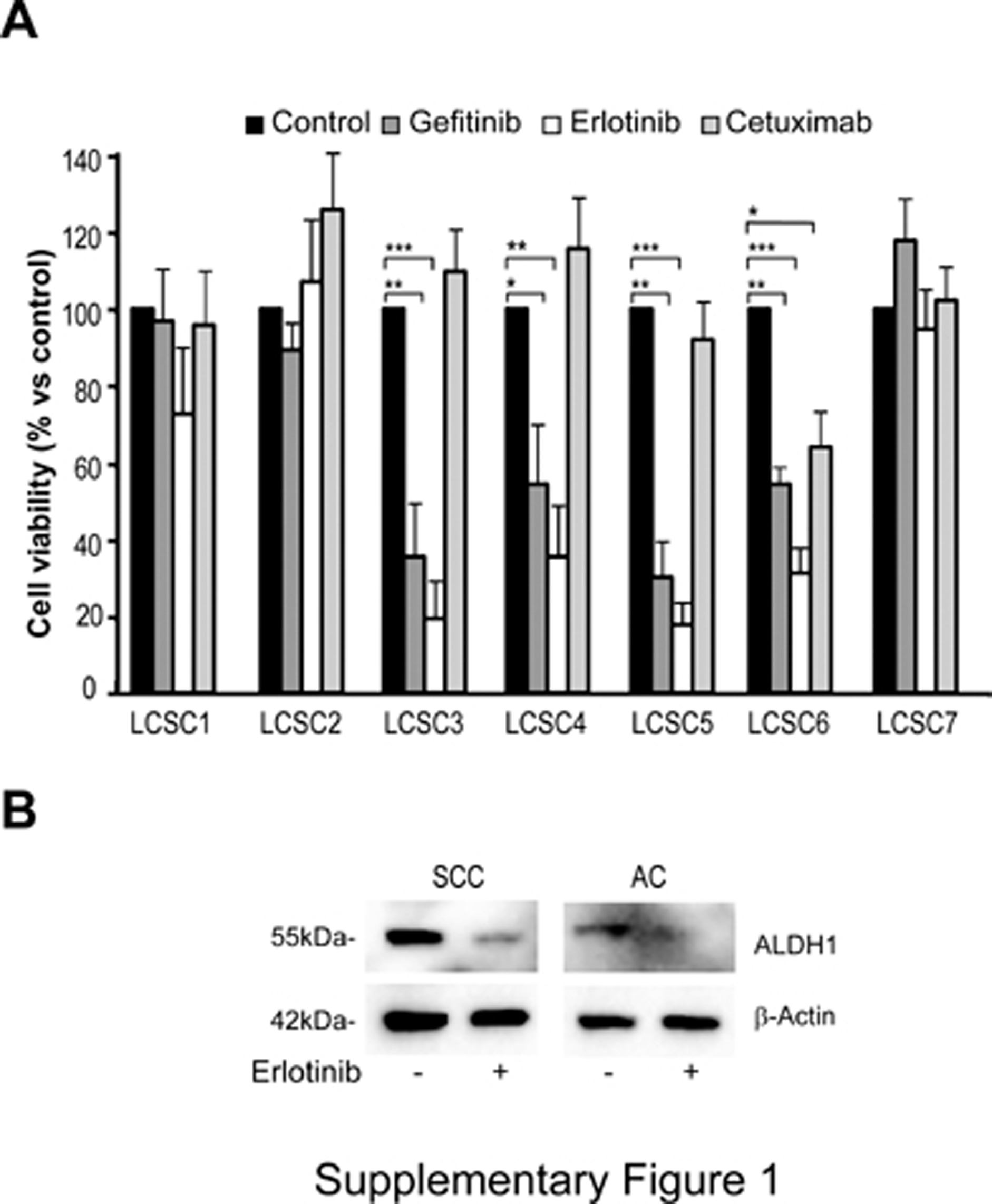

Supplement: Supplementary Figure 1 [file cddis2015217x1.tif]

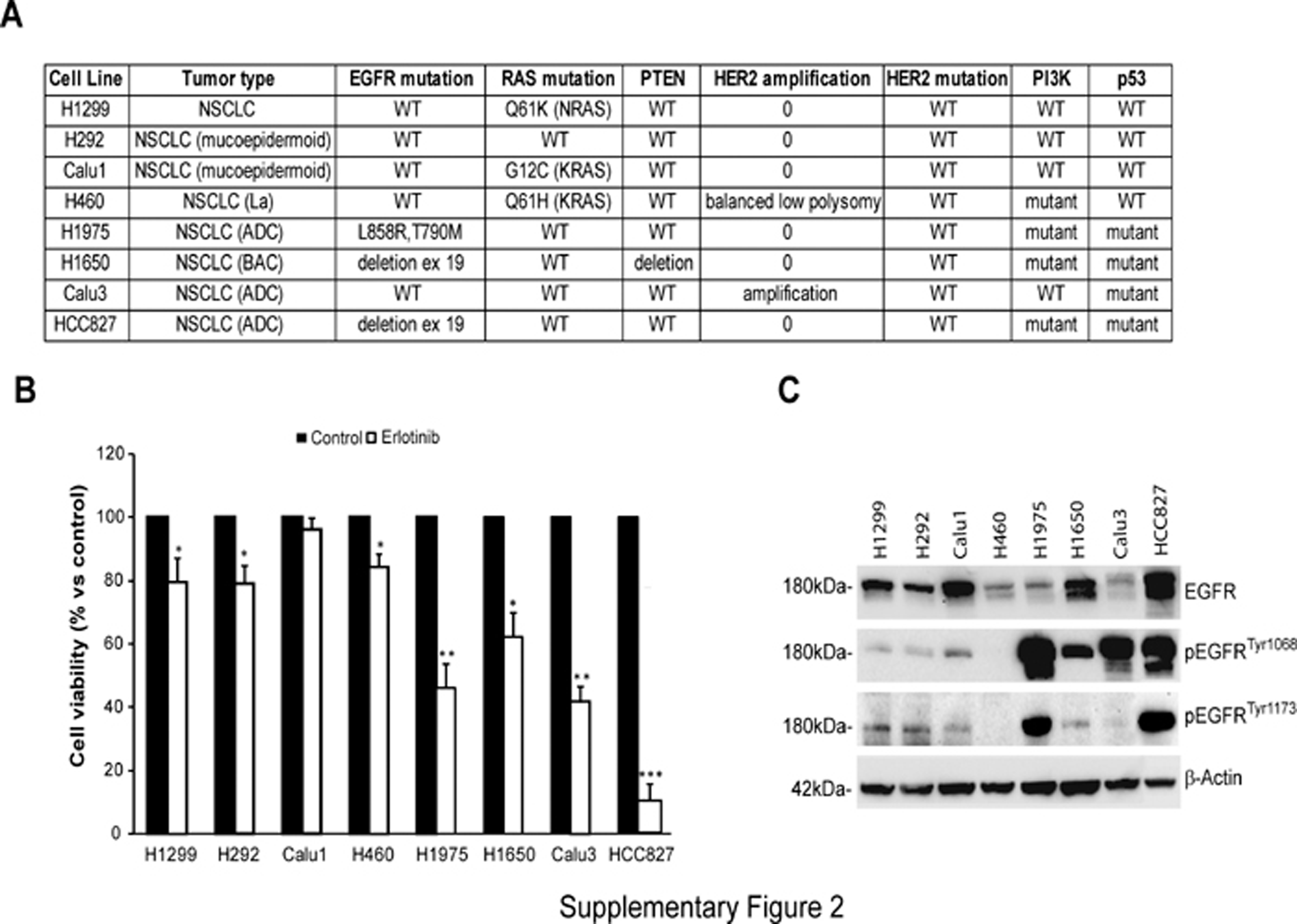

Supplement: Supplementary Figure 2 [file cddis2015217x2.tif]

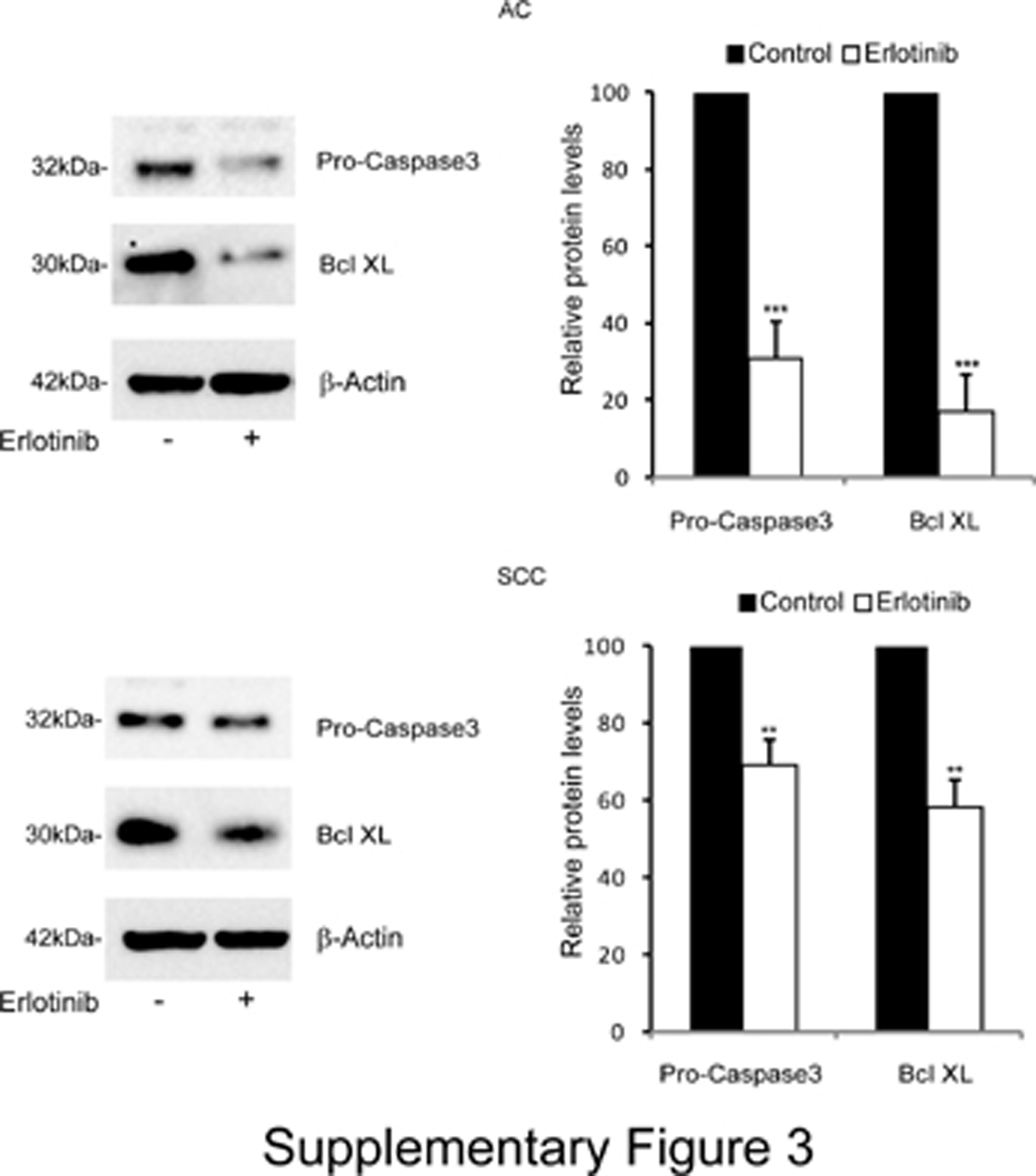

Supplement: Supplementary Figure 3 [file cddis2015217x3.tif]
