# Supplementary material for: Tyr1068-phosphorylated epidermal growth factor receptor (EGFR) predicts cancer stem cell targeting by erlotinib in preclinical models of wild-type EGFR lung cancer
Source: Cell Death Dis. 2015 Aug 6;6(8):e1850–. doi: 10.1038/cddis.2015.217 (PMC4558509; doi:10.1038/cddis.2015.217)
Supplement: Supplementary Figure Legends [file cddis2015217x4.docx]

**LEGENDS FOR SUPPLEMENTARY FIGURES**

**Supplementary Figure 1**

*A) Evaluation of EGFR inhibitors activity against lung-CSC.* Lung-CSC were exposed to IRESSA, Erlotinib or Cetuximab and cell viability was measured after 72 hours by Cell Titer Glo . Mean ± SD of 3 independent experiments is shown*.* *** p<0,001; ** p<0,01; * p<0,05. B) *Erlotinib effect on ALDH1 expression.* Representative immunoblot analysis of ALDH1 expression in control (-) or Erlotinib-treated (+) LCSC of squamous cell carcinoma (SCC) or adenocarcinoma (AC) subtype.

**Supplementary Figure 2** *EGFR inhibition, expression and activation in lung cancer cell lines with WT or mutated EGFR.* A) Tumor classification and genetic pattern of the indicated lung cancer cell lines. B) Erlotinib sensitivity of the indicated lung cancer cell lines. Cells were left untreated or exposed for 3 days to the 10μM Erlotinib and cell viability, measured by Cell Titer Glo (Promega), is indicated as percentage versus control cells. Mean ± SD of 3 independent experiments is shown*.* ** p<0,01; * p<0,05. C) Immunoblot analysis of EGFR and its phosphorylated forms in the same lung cancer cell lines as in B.

**Supplementary Figure 3** *Analysis of apoptotic pathway in control and Erlotinib-treated xenografts*

Immunoblot analysis for pro-caspase-3 and Bcl-xL in control- or Erlotinib-treated AC or SCC xenografts as indicated (left) and densitometric quantification of bands (right). Relative protein levels were quantified by using Image Lab 4.0.1 program and normalized versus β-actin as loading control.

**SUPPLEMANTARY INFORMATION**

*DNA extraction and direct sequencing.* DNA was extracted from LCSC lines (for HER2, PI3K, PTEN, KRAS) or paraffin-embedded primary and/or metastatic NSCLC (for EGFR) using the DNA extraction kit QIAmp DNA kit (Qiagen) according to the manufacturer's instructions. The concentration and purity of DNA were determined by NanoDrop 2000 Spectrophotometer (Thermo Scientific, Waltham, USA). One hundred ng of genomic DNA was used in a PCR to amplify EGFR gene (exons 19,21); *HER2* (exons 20, 21); PIK3CA (exons 10, 21); *pTEN* (exons 1 to 9). PCR conditions were as follow: 96°C for 10 min, 35 cycles of 95° for 30 sec, 55-56-58°C for 30 sec, 72°C for 30 sec and at the end 72°C for 10 minutes. Primers used are reported in Supplementary Table 1. PCR products were analyzed by electrophoresis using a 2.0% agarose gel and purified using Nucleospin Extract II Purification kit (M-Medical). Cycle sequencing was performed using BigDye Terminator v 3.1 kit (Applied Biosystems), and analyzed with a ABI 3130 capillary electrophoresis system (Applied Biosystems). K-RAS mutation (exons 2, 3,4) status was evaluated by a rapid and sensitive TaqMan assay (Entrogen, Elettrobiochimica) according to the manufacturer's protocol. The results were analyzed on Applied Biosystems 7500 Real Time PCR system using allelic discrimination assay program.

*Full lenght EGFR gene sequencing of lung-CSC.* For EGFR gene sequencing each exon was PCR amplified and PCR conditions were as follow: 96°C for 2 min, 33 cycles 96°C 30 sec, annealing temperature for 3 sec, 72°C 30sec e 72°C 10 min. Primers used and the annealing temperature for each primer couple are reported in Supplementary Table 2. Mutation analysis of the amplimers was performed via denaturing high-performance liquid chromatography (DHPLC) with the Wave 2100 System (Transgenomic) at column temperatures recommended by Navigator software, version 1.6.4.12 (Transgenomic). Amplimers with abnormal denaturing profiles were purified (Microcon PCR [Millipore]) and then sequenced bidirectionally with the ABI BigDye Terminator Sequencing Kit v.3.1

*Fluorescence in situ Hybridization* (FISH). EGFR and HER2 gene amplification and *ALK* rearrangement on chromosome 2p23 were performed by FISH, according to manufacturer's instructions (Dako) on lung-CSC after cytospin preparation. The following probes were used: EGFR/CEN-7, HER2/CEP17 (FISH pharmDx™ Kit), and ALK (FISH DNA Probe, Split Signal). Briefly, cytospin were fixed in formalin for 2 minutes and dehydrated in alcohol. FISH probes were hybridized for 18-20h at 45°C, after which the slides were washed. Mounting medium with DAPI was added and coverslips were attached to facilitate imaging. The 100X oil immersion objective was used to score signals. *Scoring criteria*. *HER2, EGFR* were defined as “amplified” (A) when the ratio between gene/CEP7 or 17 was >2. Polysomy intended as an increased CEP17/CEP7 copy number- is considered to be present when a mean number of ≥3 signals was shown. ALK gene was defined as rearranged when red and green signals are separated by ≥2 signals diameter or 3’ signals are present with no isolated 5’ signals in at least 15% of tumor cells.

*Cell lines culture and drug treatments and cell viability assay.* Lung cancer cell lines H1299, H299, Calu1, H460, H1975, H1650, Calu3, HCC827 were obtained from ATCC and grown in 10% fetal bovine serum-containing complete RPMI medium (Gibco). The cell lines were obtained directly from the ATCC that performs cell line characterizations or authentication by the short tandem repeat profiling and passaged in our laboratory for fewer than 6 months after receipt. For drug treatments and cell viability assays 3 thousand cells were plated in triplicate in 96wells plate and left untreated or exposed for 3 days to the following drugs before Cell Titer Glo evaluation (Promega): 10mM Erlotinib, 10mM Gefitinib, 100mg/ml Cetuximab.
