# Supplementary material for: Tyr1068-phosphorylated epidermal growth factor receptor (EGFR) predicts cancer stem cell targeting by erlotinib in preclinical models of wild-type EGFR lung cancer
Source: Cell Death Dis. 2015 Aug 6;6(8):e1850–. doi: 10.1038/cddis.2015.217 (PMC4558509; doi:10.1038/cddis.2015.217)
Supplement: Supplementary Table 1 [file cddis2015217x5.docx]

Supplementary Table 1

Primers used for sequencing of HER2, PI3K, PTEN and EGFR exons of interest.

| **Primers** | **Type** | **Gene** |
| --- | --- | --- |
| CCCACGCTCTTCTCACTCAT | Fw1 | *HER2* exon 20 |
| GGGTCCTTCCTGTCCTCCTA | Rew1 |  |
| CTCTCAGCGTACCCTTGTCC | Fw1 | *HER2* exon 21 |
| AGGTGCATACCTTGGCAATC | Rew1 |  |
| TTGCTTTTTCTGTAAATCATCTGTG | Fw1 | *PI3K* exon10 |
| TGCTGAGATCAGCCAAATTCAGTT | Rw1 |  |
| CATCTGTGAATCCAGAGGGGAAAA | Fw2 |  |
| GCCAAATTCAGTTATTTTTTCTGT | Rw2 |  |
| TTCTCAATGATGCTTGGCTCTGG | Fw1 | *PI3K* exon 21 |
| AATCGGTCTTTGCCTGCTGAGA | Rev1 |  |
| GCAGTGTGGAATCCAGAGTGAGC | Rev2 |  |
| TTCCATCCTGCAGAAGAAGC | Fw1 | *pTEN* exon 1 |
| CATCCGTCTACTCCCACGTT | Rw1 |  |
| ACCCCTGTGCAGTTGAAAAT | Fw1 | *pTEN* exon 2 |
| CATCACAAAGTATCTTTTTCTGTGG | Rw1 |  |
| TTTTGTTAATGGTGGCTTTTTG | Fw1 | *pTEN* exon 3 |
| AACTCTACCTCACTCTAACAAGCAGA | Rw1 |  |
| AAAGATTCAGGCAATGTTTGTT | Fw1 | *pTEN* exon 4 |
| TCTCACTCGATAATCTGGATGAC | Rw1 |  |
| TTCTGAGGTTATCTTTTTACCACA | Fw1 | *pTEN* exon 5 |
| CTCAGATCCAGGAAGAGGAAAG | Rw1 |  |
| TTTTTCAATTTGGCTTCTCTTTTT | Fw1 | *pTEN* exon 6 |
| TGGTTAAGAAAACTGTTCCAATACA | Rw1 |  |
| CAGTTAAAGGCATTTCCTGTG | Fw1 | *pTEN* exon 7 |
| TTTTGGATATTTCTCCCAATGAA | Rw1 |  |
| TGTTTAACATAGGTGACAGATTTTCTT | Fw1 | *pTEN* exon 8(a) |
| CTAGATATTCCTTGTCATTATCTGCAC | Rw1 |  |
| TATGTGATCAAGAAATCGATAGCA | Fw1 | *pTEN* exon 8(b) |
| ACATACATACAAGTCAACAACCC | Rw1 |  |
| TTGTGGGTTTTCATTTTAAATTTTC | Fw1 | *pTEN* exon 9 |
| AAAAGGTCCATTTTCAGTTTATTCA | Rw1 |  |
| GTCACAGCCCCCAGCAATAT | Fw2 |  |
| GATGTGGAGATGAGCAGGGT | Rw2 |  |
| CCAGATCACTGGGCAGCATGTGGCACC | Fw1 | EGFR exon 19 |
| CTAGAGCAGAGCTGCC | Rw1 |  |
| GTCACAGCCCCCAGCAATAT | Fw2 |  |
| GATGTGGAGATGAGCAGGGT | Rw2 |  |
| TCAGAGCCTGGCATGAACATGACCCTG | Fw1 | EGFR exon 21 |
| CTGGTCCCTGGTGTCAGGAAAATGCTGG | Rw1 |  |
| TTCATGCGCCTTTCCATTCTTT | Fw2 |  |
| CTGGTCCCTGGTGTCAGGAAA | Rw2 |  |
