# Supplementary material for: Tyr1068-phosphorylated epidermal growth factor receptor (EGFR) predicts cancer stem cell targeting by erlotinib in preclinical models of wild-type EGFR lung cancer
Source: Cell Death Dis. 2015 Aug 6;6(8):e1850–. doi: 10.1038/cddis.2015.217 (PMC4558509; doi:10.1038/cddis.2015.217)
Supplement: Supplementary Table 2 [file cddis2015217x6.docx]

| **Primers (annealing T)** | **Type** | **Exon** |
| --- | --- | --- |
| ACGCCACAACCACCGCGCAC (64°) | Fw1 | *EGFR* exon 1 |
| GGCGCAGCTGATCTCAAGGA | Rew1 |  |
| GAGTCTCTGTGTGGAGAGAG (60°) | Fw1 | *EGFR* exon 2 |
| AGACTTGTCATTGCCATAGC | Rew1 |  |
| GAGCACTCGTGTGCATTAGG (60°) | Fw1 | *EGFR exon 3* |
| ATTGAGTGACAAGCTCGCTG | Rw1 |  |
| TGCGAAGAGCACATGCATCC (60°) | Fw1 | EGFR exon 4 |
| TGTCCGTGGTAAATACATGC | Rw1 |  |
| AGTGATTCTACAAACCAGCC (60°) | Fw1 | *EGFR exon 5* |
| CTGGTTCACATCTGACCCTG | Rev1 |  |
| CGTTGGAAGCAAATGTGTCT (60°) | Fw1 | *EGFR exon 6* |
| GGAAGTCTTCTGTCCTGGTG | Rw1 |  |
| GACAGTAACTTGGGCTTTCT (60°) | Fw1 | *EGFR exon 7* |
| TCTCCAAGATGGGATACTCC | Rw1 |  |
| GAACGGGATTCCTTCTCTAT (58°) | Fw1 | *EGFR exon 8* |
| TCCTTTGGAGGTGGCATGAG | Rw1 |  |
| GACCGGAATTCCTTCCTGCT (60°) | Fw1 | *EGFR exon 9* |
| TGATCACCAATCTCTACCAG | Rw1 |  |
| CTGGTAGAGATTGGTGATCA (60°) | Fw1 | *EGFR exon 10* |
| AGCGTGTTTACACCGCAGTG | Rw1 |  |
| GAGAGTCTAGAGTAATGTCTCA (60°) | Fw1 | *EGFR exon 11* |
| GTGCCCTATCTTAGCAACTC | Rw1 |  |
| TGCAGTGTGTGCCTCCCACA (60°) | Fw1 | *EGFR exon 12* |
| GACCCAGTTAGAACCAACTCC | Rw1 |  |
| AGCCAGCATGTCTGTGTCAC (60°) | Fw1 | *EGFR exon 13* |
| GCAATCAAACAACTTCCCGTC | Rw1 |  |
| GTCCTGGAGTCCCAACTCCT (60°) | Fw1 | *EGFR exon 14* |
| TCTGGGAAGTGGCTCTGATG | Rw1 |  |
| GGTGCAATCACAGAATAACTG (60°) | Fw1 | *EGFR exon 15* |
| ACTCAAATACAAACCTCGGC | Rw1 |  |
| CCACCAATCCAACATCCAGA (60°) | Fw1 | EGFR exon 16 |
| ACTTGATCAGGACAGAGGAC | Rw1 |  |
| CTACAAGATGTCAGTGCACT (60°) | Fw1 | EGFR exon 17 |
| CTGGGTATAACTGCACATTC | Rw1 |  |
| CTGTGTGTGTCACTCGTAAT (60°) | Fw1 | EGFR exon 22 |
| CATTGCAATCTCCAGTGACT | Rw1 |  |
| CCAGCATTGAAGCAAATTGC (60°) | Fw1 | EGFR exon 23 |
| TTGGCTAAGAGCAGCCACCA | Rw1 |  |
| GTACTAGCTGGCCAAGACAG (60°) | Fw1 | EGFR exon 24 |
| TGGCATGTGACAGAACACAG | Rw1 |  |
| GCTGGCAATAGACCCCTGCT (58°) | Fw1 | EGFR exon 25 |
| GTTCAAATGAGTAGACACAGC | Rw1 |  |
| CACCTTCACAATATACCCTC (62°) | Fw1 | EGFR exon 26 |
| CTGTGAGGCGTGACAGCCGT | Rw1 |  |
| TCAGGCCTGCCCAACCTACT (64°) | Fw1 | EGFR exon 27 |
| GCTTGGACACTGGAGACTGG | Rw1 |  |
| CACAGGGTTCAGAACCCAGG (64°) | Fw1 | EGFR exon 28 |
| AGAGCTAATGCGGGCATGGC | Rw1 |  |

Supplementary Table 2

Primers used for sequencing of EGFR.
